# Spatial Cluster of Air Pollutants and Its Association with Life Expectancy, Age-Specific Mortality Risk, and Cause-Specific Mortality Rate: A County-Level Ecological Study Across the USA

**DOI:** 10.3390/life16010177

**Published:** 2026-01-21

**Authors:** Jing Wang, Qiaochu Xu, Rong Rong, Bingjie Qu, Xiang Shi, Bin Hu, Peng Zhao, Chengxiu Ling, Ying Chen

**Affiliations:** 1 Wisdom Lake Academy of Pharmacy, Xi’an Jiaotong-Liverpool University, Suzhou 215123, China; 2 Department of Environmental Health, Harvard School of Public Health, Boston, MA 02115, USA; 3 Department of Health Policy and Management, School of Public Health and Tropical Medicine, Tulane University, New Orleans, LA 70112, USA; 4 Institute of Systems, Molecular & Integrative Biology, University of Liverpool, Liverpool L69 7BE, UK; 5 Department of Biology, School of Science, Xi’an Jiaotong-Liverpool University, Suzhou 215123, China; 6 Key Lab for Biomedical Effects of Nanomaterials and Nanosafety, Chinese Academy of Sciences, National Center for Nanoscience and Technology, Beijing 100190, China; 7 Department of Health and Environmental Sciences, School of Science, Xi’an Jiaotong-Liverpool University, Suzhou 215123, China

**Keywords:** air pollution, health inequality, geographical pattern, latent class analysis, epidemiology

## Abstract

Air pollution has been one of the major threats to public health. The study aimed to determine latent patterns of geographical distribution of health-related air pollutants across the USA and to evaluate real-world cumulative effects of these patterns on public health metrics. It was an ecological study using county-level data on the concentrations of 12 air pollutants over 20 years. Latent class analysis was used to identify the common clusters of life expectancy-associated air pollutants based on their concentration characteristics in the final counties studied (*n* = 699). Multivariate linear regression analyses were then applied to assess the relationship between the LCA-derived clusters and health measurements with confounding adjustment. We found that PM_2.5_ mass, PM_10_ speciation, and NONO*_x_*NO*_y_* (the reactive nitrogen species) were associated with life expectancy and thus were included in LCA. Among five identified clusters, the one with a more severe pollutant profile was associated with a decreasing life expectancy, an increasing mortality risk among middle-aged and elderly populations (≥45 years), and an increasing mortality rate caused by chronic respiratory conditions, cardiovascular diseases, and neoplasms. Our study brings new perspectives on real-world geographical patterns of air pollution to explain health disparities across the USA.

## 1. Introduction

Air pollution is a complicated mixture composed of gaseous pollutants, particulates, and other toxic pollutants such as hazardous air pollutants (HAPs), particularly arsenic, volatile organic compounds (VOCs), and lead. It represents one of the major global threats to environmental sustainability and public health, contributing to over 4 million deaths annually [1]. Generally, exposure to air pollution is associated with oxidative stress and inflammation in human cells, which may lay a foundation for a variety of acute and chronic diseases [2,3,4,5,6,7,8]. Abundant research illustrates that air pollution could lead to adverse health consequences, including respiratory and cardiovascular disease, neurological disorders, adverse birth outcomes, and cancer [9,10,11,12,13,14,15,16].

Air pollutants such as ozone, carbon monoxide (CO), nitrogen dioxide (NO_2_), sulfur dioxide (SO_2_), particulate matter (PM_2.5_ and PM_10_), HAPs, VOCs, nitric oxide (NO), nitrogen oxides (NO*_y_*), and lead contribute to significant adverse health impacts across multiple biological systems. Ozone is widely studied for its association with respiratory health impacts, including asthma exacerbation, chronic obstructive pulmonary disease (COPD), and increased mortality rates [17,18,19]. CO is particularly associated with elevated cardiovascular risks and adverse postoperative outcomes [20,21,22,23,24]. NO_2_ and NONO*_x_*NO*_y_* (referring to the reactive nitrogen species) are associated with increased respiratory diseases and also linked to neurological and mental health disorders due to their inflammatory and oxidative properties [25,26]. SO_2_ adversely affects respiratory function and is implicated in various respiratory diseases [27,28,29]. PM_2.5_ and PM_10_ contribute substantially to respiratory illness, cardiovascular diseases, neurological disorders, and overall mortality due to deep lung penetration and systemic inflammation [30,31,32]. HAPs and VOCs, including benzene, toluene, ethylbenzene, and xylene (BTEX), significantly elevate cancer risks and reproductive and developmental problems, including infertility and epigenetic modifications [33,34,35,36,37]. Lead exposure critically affects neurological health and cognitive development [26,38,39]. Collectively, these pollutants were acknowledged as representing a comprehensive set for assessing air pollution exposure impacts on population health [40].

Notably, particulate matter with aerodynamic diameter ≤ 2.5 μm (PM_2.5_) and ≤ 10 μm (PM_10_) are critical components of air pollution. Both PM_2.5_ and PM_10_ could be inhaled and deposited through people’s airways. Compared to PM_10_, which tends to deposit on the larger airways of the upper region of the lung, PM_2.5_ is usually deposited on deeper regions of the respiratory tract and is easily absorbed into the body through the bloodstream, olfactory nerves, and gastrointestinal system due to its smaller size [41]. Long-term exposure to PM_2.5_ is associated with premature death and reduced life expectancy, increased risks of cardiometabolic diseases, asthma, reduced visibility, and other detrimental health effects [42,43]. Similarly, long-term exposure to ambient PM_10_ is linked to reduced lung function, elevated hospitalization, and emergency department visits for respiratory and cardiovascular diseases, including asthma, chronic obstructive pulmonary disease (COPD), high blood pressure, heart attack, and strokes [41]. In addition to particulate matter, nitrogen oxides, a mixture of gases consisting of nitrogen and oxygen, particularly nitrogen dioxide (NO_2_) and nitric oxide (NO), are another important type of air pollutant. Long-term NO_2_ is associated with elevated all-cause, circulatory, ischemic heart disease, respiratory, and lung cancer mortality, and acute lower respiratory infections [44]. NO exposure is associated with respiratory syncytial virus infection, neurodegeneration, and diseases related to the nervous system [45,46,47].

Associations between individual air pollutants and specific health outcomes are well established in the current literature. However, a more systematic understanding of the spatial distribution of simultaneous exposure to various health-related air pollutants across large geographical areas and their cumulative influence on overall health disparities remains limited. Previous research found that exposure to high levels of ozone, PM_2.5_, and NO_2_ simultaneously was associated with impaired lung function and an increased incidence of cardiovascular conditions [48,49]. Higher concentrations of PM_2.5_, NO, and NO_2_ were associated with a higher risk of low birth weight [50]. Young populations exposed to high levels of PM_2.5_, PM_10_, SO_2_, and nitrogen oxides simultaneously were more likely to develop phlegm, bronchitis, and asthma [51]. However, the selection of studied air pollutants was based on the availability of data rather than on a systematic approach that could identify pollutants relevant to health.

Therefore, in this study, we hypothesized that co-exposure to particulate matter and nitrogen oxides was associated with the measurements of overall health. We first aimed to systematically identify the air pollutants that are most closely associated with life expectancy from those listed by the Environmental Protection Agency (EPA) using the national data of the USA. Second, we aimed to use cluster analysis to determine the common latent patterns of the geographical distribution of these life expectancy-associated air pollutants. Finally, using these derived patterns, we aimed to evaluate the associations between real-world collective air pollutants and the measurements of overall health, including life expectancy at birth, age-specific mortality risks, and cause-specific mortality rates. Our analysis was structured to explore cumulative health impacts within defined clusters, reflecting real-world multi-pollutant exposure scenarios. This design was essential to address existing gaps in understanding how combined pollutant exposures relate spatially to health disparities across diverse regions.

## 2. Materials and Methods

This is an ecological study with a county or county equivalent (e.g., independent cities, parishes in Louisiana, and the District of Columbia) as the sample unit. The USA county-level air pollutants data and rigid public health metrics, including life expectancy at birth, age-specific mortality risks, and cause-specific mortality rates, were analyzed. For potential confounding adjustment, characteristics of the population, socioeconomics, healthcare service, and residential environment and location data were also collected.

### 2.1. Database and Variable

Daily records of twelve air pollutants within three categories, which are criteria gases (including ozone, CO, NO_2_, and SO_2_), particulates (including PM_2.5_ mass, PM_2.5_ speciation, PM_10_ mass, and PM_10_ speciation), and toxics, precursors, and lead (including HAPs, VOCs, NONO*_x_*NO*_y_*, and lead), at the county level between 1995 and 2014 (in total 20 years) were obtained from the USA Environmental Protection Agency (https://aqs.epa.gov/aqsweb/airdata/download_files.html#Annual (accessed on 14 August 2024)) [52]. Technical information about the measured parameters, units, and classification of these air pollutants is shown in Table S1 in the Supporting Information [53]. Notably, we included both the mass and speciation of particulate matter (PM) to investigate its potential health effects as a whole or the health effects associated with specific components. PM mass refers to the total mass concentration of PM, and PM speciation refers to the multiple components of PM [54]. At least 1 of the 12 air pollutants was monitored in 1388 counties between 1995 and 2014. Annual average concentrations of the studied air pollutants from 1995 to 2014 were calculated based on the daily data. Counties without an air pollution measurement were not included in our analysis. The median number of air quality monitors within a studied county was 2, and where there were multiple monitors within a county, we used the average concentration to summarize the overall pollution level in that county.

County-level health metrics of the residential population in this study included life expectancy at birth, age-specific mortality risks, and 21 mutually exclusive age-standardized cause-specific mortality rates in 2014, which directly came from the Institute for Health Metrics and Evaluation (IHME). Specifically, the life expectancy at birth and age-specific mortality risks were estimated using small area estimation methods, which produce annual county-level life tables. These estimates utilized de-identified death records from the National Center for Health Statistics (NCHS), and population counts from the Census Bureau, NCHS, and the Human Mortality Database [55]. The age-specific mortality risks were presented in the following age categories: 0–4, 5–24, 25–44, 45–65, and 65–84 years old [56]. For cause-specific mortality rates, redistribution of garbage codes and small area estimation methods were used on National Vital Statistics System data to estimate annual county-level mortality rates for 21 causes of death [57]. The 21 mutually exclusive causes of death were divided into three groups: communicable, maternal, neonatal, and nutritional diseases; noncommunicable diseases; and injuries. Communicable, maternal, neonatal and nutritional diseases included (1) HIV/AIDS and tuberculosis; (2) diarrhea, lower respiratory, and other common infectious diseases; (3) neglected tropical diseases and malaria; (4) maternal disorders; (5) neonatal disorders; (6) nutritional deficiencies; and (7) other communicable, maternal, neonatal, and nutritional diseases. Noncommunicable diseases included (8) neoplasms; (9) cardiovascular diseases; (10) chronic respiratory diseases; (11) cirrhosis and other chronic liver diseases; (12) digestive diseases; (13) neurological disorders; (14) mental and substance use disorders; (15) diabetes, urogenital, blood, and endocrine diseases; (16) musculoskeletal disorders; (17) other non-communicable diseases. Injuries included (18) transport injuries; (19) unintentional injuries; (20) self-harm and interpersonal violence; and (21) forces of nature, war, and legal intervention [58].

County-level information on population characteristics (including size, gender, age, and ethnicity), socioeconomics (including educational level, annual median household income, unemployment rate, and poverty rate), healthcare service (including medically insured rate and the number of physicians per 1000 population), and residential environment and location (including Rural Urban Continuum Code, latitude, and longitude) were collected from the USA national official sources as potential confounders and relevant covariates in the statistical analysis (Table S2 in the Supporting Information) [59,60,61,62,63,64,65,66].

### 2.2. Statistical Approach and Analysis

We used the mean concentrations over 20 years (from 1995 to 2014) to measure previous long-term exposure to air pollutants and evaluate their relationship with life expectancy in 2014. Initial multiple linear regression analyses, controlling for all covariates listed above, were conducted separately for the 12 individual air pollutants to identify potential candidates of risk factors. Multiple linear regression models with the backward selection method were then used to identify the final list of significant air pollutants associated with life expectancy, controlling the same variables. In this approach, a five-time repeated ten-fold cross-validation resampling scheme was carried out to assess the performance of the model via obtaining the mean squared error, without a high potential for biased estimation [67,68]. This method was implemented 20 times, and the final set of air pollutants enrolled for later cluster analysis was those significantly associated with life expectancy at each time of the optimal model.

After the final list of life expectancy-associated air pollutants was determined, our study samples (USA counties) included in later cluster analysis were identified based on the following criteria: a county containing one unmonitored pollutant (missing data) at most from the final listed air pollutants. Where there were missing data, multiple imputation using the predictive mean matching method was implemented according to the standard approach through an available package in R (the ‘mice’ package) [69].

Latent cluster analysis (LCA) was used to identify the common distribution patterns of the air pollutants across the USA. LCA-clustered counties were determined by the concentration features of the final listed air pollutants derived from the previous selection process. Before entry into LCA, data of air pollutant concentrations were classified into three categories (‘low,’ ‘medium,’ and ‘high,’ ordinal data) according to the 33rd and 67th percentiles of their twenty-year mean concentrations. We reported log-likelihood (LL) statistics with bootstrap *p*-values, Bayes Information Criterion, and Consistent Akaike’s Information Criterion for each model containing cluster numbers from 1 to 10. LCA was carried out in Latent GOLD (version 4.5) with Newton–Raphson algorithms and estimation-maximization being utilized for model parameter estimation [70]. One thousand different random starting values were applied, and each included 50 interactions. Bootstrap *p*-values were determined to assess the model fit based on the LL statistics. The optimal model is the one with the largest number of clusters where the *p*-value remains significant at the desired significance level (5%). Each county was allocated into one cluster according to its posterior probabilities of belonging to each cluster. A mean posterior probability ≥ 0.7 for samples allocated to a cluster was considered a good assignment [71].

Multiple linear regression models were then developed to assess the associations between the LCA-derived clusters and life expectancy (2014 data), change in life expectancy (from 1995 to 2014), age-specific mortality risk (2014 data), and cause-specific mortality rate (2014 data), with adjustment for potential confounding factors including collected information on population characteristics, socioeconomics, healthcare service, and residential environment and location. Our statistical analysis approaches have been applied to other studies previously [72,73].

All statistical analyses were performed in R (version 4.0.4) except for LCA. To correct the effect of multiple testing, a *p*-value < 0.005 (instead of 0.05), two-tailed, was set as the threshold for statistical significance, aiming to obtain conservative results with a low level of false-positive findings. The flowchart of database construction and statistical analysis is shown in Figure S1 in the Supporting Information.

## 3. Results

Initial analyses showed that concentrations of ozone, PM_2.5_ mass, PM_10_ mass, PM_10_ speciation, and NONO*_x_*NO*_y_* were associated with life expectancy, whereas the other studied air pollutants (SO_2_, CO, NO_2_, PM_2.5_ speciation, VOCs, HAPs, and lead) were not. Multiple linear regression analyses with the backward selection method and a five-time repeated ten-fold cross-validation resampling scheme further indicated that only PM_2.5_ mass, PM_10_ speciation, and NONO*_x_*NO*_y_* were suggestive of a consistent association with life expectancy (Table S3 in the Supporting Information), which determined these three air pollutants to be included in the subsequent analyses.

Descriptive statistics of the final studied counties (*n* = 699) regarding the concentrations of the three selected air pollutants, health measures, and socio-demographic variables are shown in Table 1. The geographical distribution of these counties is displayed in Figure S2 in the Supporting Information, informing generally representative samples at the USA national level.

In LCA, the five-cluster model was determined as the optimal model (Table S4 in the Supporting Information). Studied counties generally displayed high posterior probabilities for their assigned clusters, with mean posterior probabilities ranging from 0.66 to 0.93 across the five clusters (Table 2).

**Figure 1:**
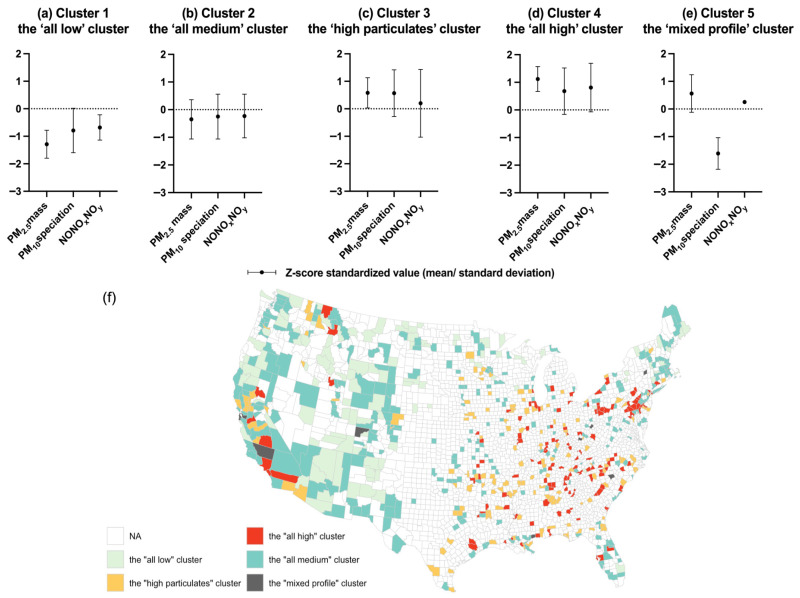
Concentration characteristics of PM_2.5_ mass, PM_10_ speciation, and NONO*_x_*NO*_y_* by latent class analysis-derived clusters and their spatial distribution across the USA. (**a**) Cluster 1—the ‘all low’ cluster, (**b**) Cluster 2—the ‘all medium’ cluster, (**c**) Cluster 3—the ‘high particulates’ cluster, (**d**) Cluster 4—the ‘all high’ cluster, (**e**) Cluster 5—the ‘mixed profile’ cluster, subfigure (**f**) is the spatial distribution of the pollutant concentration patterns as the highlight.

Cluster 1 (*n* = 115, 16.5%) was featured with low concentrations of PM_2.5_ mass, PM_10_ speciation, and NONO*_x_*NO*_y_* (the ‘all low’ cluster, Figure 1a). Cluster 2 (*n* = 285, 40.8%), the most common cluster, displayed medium levels of all three air pollutants (the ‘all medium’ cluster, Figure 1b). Cluster 3 (*n* = 152, 21.8%) was characterized by high PM_2.5_ mass and PM_10_ speciation (the ‘high particulates’ cluster, Figure 1c), whereas Cluster 4 (*n* = 136, 19.5%) had the highest levels of all three air pollutants (the ‘all high’ cluster, Figure 1d). Cluster 5, with the smallest size (*n* = 11, 1.6%), displayed a mixed profile: high PM_2.5_ mass but low PM_10_ speciation (the ‘mixed profile’ cluster, Figure 1e).

The geographical distribution of the counties stratified by the five LCA-derived clusters is presented in Figure 1f. Counties in Cluster 1, characterized by low air pollutant concentrations, were primarily located in the west and northeast regions. Most counties included in Clusters 3 and 4, which had relatively high pollution levels of the three air pollutants, were found in the Midwest, the South, and the southern part of the Pacific regions. Counties classified into Cluster 2, with medium levels of all air pollutants, were distributed widely across the whole USA, whereas the distribution pattern of Cluster 5 could not be summarized due to its small sample size.

Table S5 in the Supporting Information shows the county-level descriptive statistics of the three air pollutants and health outcome measurements stratified by the five clusters. Average county-level life expectancy in Cluster 1 was the highest (79.33 years, standard deviation (SD) 1.84), while Cluster 4 was the lowest (77.40 years, SD 2.03). In multivariate analysis, compared to Cluster 1, Clusters 2, 3, and 4 were all significantly associated with a reduced county-level life expectancy at birth, with adjustment for collected potential confounding factors (left, Table 3). Similar results were obtained for life expectancy change as the outcome variable; however, only Clusters 3 and 4 reached statistical significance (right, Table 3).

**Figure 2:**
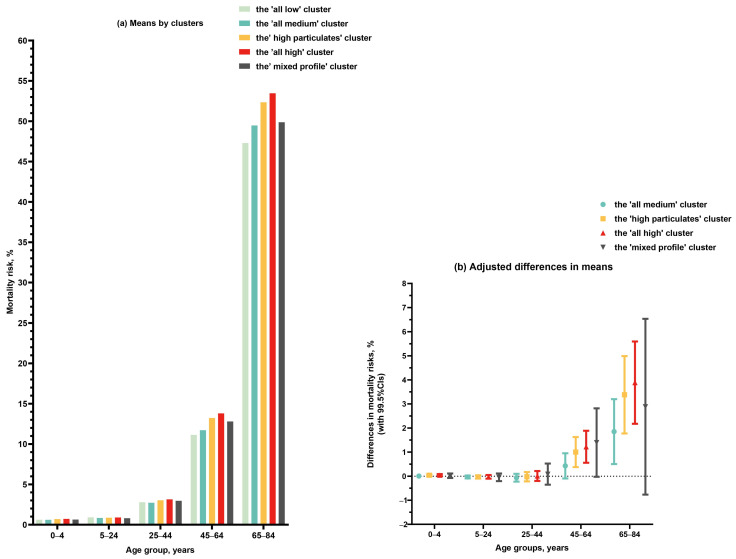
Age-specific mortality risk by latent class analysis-derived clusters of air pollutants. The ‘all low’ cluster (cluster 1) was the referent group; all models adjusted for population characteristics (including size, gender, and ethnicity), socioeconomics (including educational level, annual median household income, unemployment rate, and poverty rate), healthcare service (including medical insured rate and the number of physicians per 1000 population), and residential environment and location (including Rural–Urban Continuum Codes, latitude, and longitude).

Differences in mortality risks were seen between these LCA-derived clusters, specifically among middle-aged and elderly populations (Figure 2a). For example, for the group of 45–64 years, the mortality risks were 11.14% (SD 2.03%), 11.71% (SD 2.62%), 13.23% (SD 2.94%), 13.80% (SD 2.71%), and 12.80% (SD 3.56%) for those who lived in the counties of ‘all low’ (Cluster 1), ‘all medium’ (Cluster 2), ‘high particulates’ (Cluster 3), ‘all high’ (Cluster 4) and ‘mixed profile’ (Cluster 5) clusters, respectively (Table S5 in the Supporting Information). The confounding-adjusted difference in age-specific mortality risks between clusters is shown in Figure 2b (the ‘all low’ Cluster 1 as the referent group), indicating consistently increased mortality risks among middle-aged and elderly populations in severely polluted regions.

**Figure 3:**
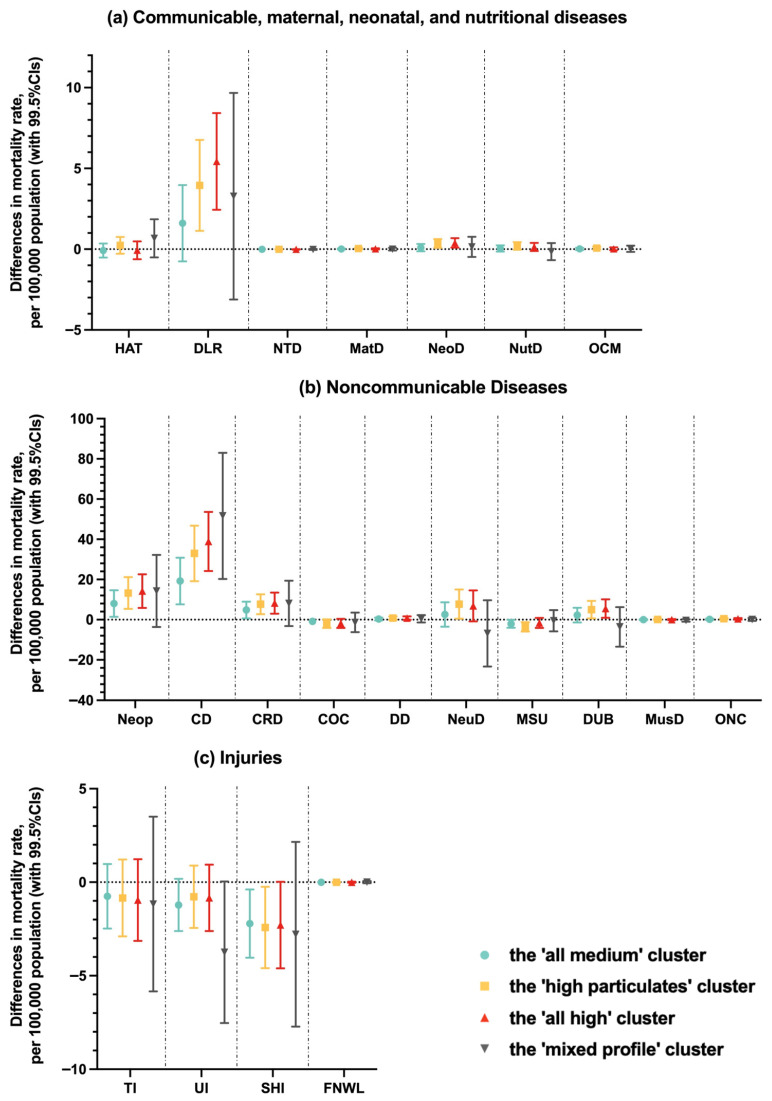
Cause-specific mortality rate by latent class analysis-derived clusters of air pollutants The ‘all low’ cluster (cluster 1) was the referent group; all models adjusted for population characteristics (including size, gender, and ethnicity), socioeconomics (including educational level, annual median household income, unemployment rate, and poverty rate), healthcare service (including medical insured rate and the number of physicians per 1000 population), and residential environment and location (including Rural–Urban Continuum Codes, latitude, and longitude). Disease group: HAT: HIV/AIDS and tuberculosis; DLR: diarrhea, lower respiratory and other common infectious diseases; NTD: neglected tropical diseases and malaria; MatD: maternal disorders; NeoD: neonatal disorders; NutD: nutritional deficiencies; OCM: other communicable, maternal, neonatal, and nutritional diseases; Neop: neoplasms; CD: cardiovascular diseases; CRD: chronic respiratory diseases; COC: cirrhosis and other chronic liver diseases; DD: digestive diseases; DeuD: neurological disorders; MSU: mental and substance use disorders; DUB: diabetes, urogenital, blood, and endocrine diseases; MusD: musculoskeletal disorders; ONC: other non-communicable diseases; TI: transport injuries; UI: unintentional injuries; SHI: self-harm and interpersonal violence; FNWL: forces of nature, war, and legal intervention.

For cause-specific mortality rates, health conditions associated with these LCA-derived clusters were neonatal disorders, diarrhea, lower respiratory, and other common infectious diseases (in the communicable, maternal neonatal, and nutritional disease category, Figure 3a), neoplasms, cardiovascular diseases, and chronic respiratory diseases (in the noncommunicable disease category, Figure 3b), and self-harm and interpersonal violence (in the injury category, Figure 3c). In general, results suggested that clusters with more severe pollution were associated with increased mortality rates of these conditions, except for self-harm and interpersonal violence.

## 4. Discussion

Epidemiological research has typically been conducted only on a single air pollutant and related diseases due to difficulties in the design, cost, and management of a single study containing a comprehensive list of air pollutants and grand indicators of public health. Using recently available high-quality open-access databases, our ecological study at the USA national scale has made an effort to evaluate the associations between real-world multiple air pollutants and human health and could provide a novel perspective to explain the existing health disparities and a hint at future policy planning and resource allocation regarding the prevention and treatment of air pollution.

In this study, regional concentrations of air pollutants, including PM_2.5_ mass, PM_10_ speciation, and NONO*_x_*NO*_y_*, were found to be associated with life expectancy, which was consistent with our hypotheses and previous research. It is also noticeable that PM_2.5_ mass, rather than PM_2.5_ speciation, and PM_10_ speciation, rather than PM_10_ mass, were selected as life expectancy-associated air pollutants. It might imply the nuanced differences in the underlying mechanisms of how particulate matter with different sizes could impact population health. It is possible that the total mass concentration of PM_2.5_ as a whole is readily absorbable into human bodies, causing oxidative stress, inflammation, and potential other lesions in the tissues through the bloodstream, olfactory nerves, and gut [74]. However, PM_10_ itself as a whole might be too large for body absorption. Instead, major components of PM_10_, for instance, certain metal elements and soluble ions, are more easily inhaled and could penetrate deeply into the tissues and vessels, potentially leading to or exacerbating respiratory and cardiovascular diseases [75]. Future studies are needed to clarify the reasons underlying the differing impacts of PM mass and its various components.

Using LCA, we identified five clusters with distinct profiles of three life expectancy-associated air pollutants, which together depict the spatial distribution patterns of critical air pollutants across the USA. Some previous research that adopted a similar type of cluster analysis approach also identified air pollutant clusters in various geographic regions. For instance, a study found that pollutant clusters with relatively higher levels of PM_2.5_, NO_2_, and NO demonstrated an elevated risk of term low birth weight within Los Angeles County [50]. Populations living in municipalities within clusters with the highest concentrations of particulate matter, including both PM_2.5_ and PM_10_, exhibited significantly elevated excess mortality from COVID-19 in the Lombardy region of Italy [76]. Similar to the results of our studies, clusters with higher concentrations of particulate matter and NO_x_ were associated with adverse population health outcomes. However, although some previous studies have described the spatial distributions and characteristics of specific air pollutants separately, as well as their associations with specific health outcomes, none have considered the patterns of multiple air pollutants in a relatively comprehensive way, particularly in relation to several indicators of population health [77,78,79,80,81].

Further assessment of the relationship between these clusters and health measurements revealed that Clusters 3 (the ‘high particulates’ cluster) and 4 (the ‘all high’ cluster) were associated with lower life expectancy, higher age-specific mortality risks among middle-age and elderly populations, and higher rates of mortality caused by several diseases including those with the greatest burden (i.e., neoplasms, cardiovascular diseases, and chronic respiratory diseases). It was noticeable that the association of more severely polluted clusters with increased mortality risks was only seen among middle-aged and elderly populations but not in younger people. This finding implies that the possible associations of prolonged exposure to these air pollutants and adverse health outcomes are very likely to be a slow and cumulative process. Previous studies provided varying degrees of evidence for the effects of these individual air pollutants on respiratory and cardiovascular systems and cancer [5,82,83,84,85,86]. However, our study advances this research area by looking at the real-world patterns of multiple air pollutants within a geographical region and investigating the associations between exposure to collective air pollutants and different diseases.

Disparities in air pollution in different regions have been a constant phenomenon across the USA. Previous studies suggested that the Midwest, East South Central, and California states were confronted with high concentrations of PM_2.5_, while the western regions had a medium level of PM_2.5_ pollution [78]. Consistent with these findings, the spatial distribution pattern of studied air pollutants, as demonstrated in our study, indicated that the clusters with the highest levels of PM_2.5_ mass (i.e., Clusters 3 and 4) were mainly located in the Midwest, south, and south of the Pacific region, whereas the cluster with a medium level of PM_2.5_ mass (i.e., Cluster 2) showed a dense presence in the west of the country. However, regarding the distribution of PM_10_ speciation, a previous study found that the PM_10_ level in the western part of the USA was relatively higher than the rest of the regions between 1985 and 2000, based on the geographical information system-based estimators [79], which is slightly inconsistent with our identified patterns: most counties within Clusters 3 and 4 were gathered in the Midwest and south of the USA, though both studies found that the concentration of PM_10_ in the south of the Pacific region was high. This variety might be due to the different measurement methods and study periods between the two studies. Furthermore, according to our study, Cluster 4, with the highest level of NONO*_x_*NO*_y_*, also appeared in California, which supports another study conducted in Los Angeles demonstrating a particularly high regional level of NO [50]. Large cities with denser populations are accompanied by more transportation emissions, more intense economic activities releasing air pollutants, and thus more severe air pollution [87]. Our results demonstrated that many of these regions, such as the northeast metropolitan area, were classified as the ‘all high’ cluster (Cluster 4).

In the previous literature, various research has demonstrated the substantial geographical disparities in life expectancy across the USA. For example, based on the data of 2014, the life expectancy at birth was generally low in the counties located in the middle east and southeast areas, such as the south region of Mississippi, western Virginia, and eastern Kentucky, but it was the highest in Middle Colorado [55]. The former areas overlapped with the counties classified into Clusters 3 and 4 analyzed in this study, whereas a considerable number of counties in Colorado were allocated to Cluster 1. Differences in life expectancy should refer to disparities in the occurrence and outcome of diseases. Previous research indicated that the southeast and middle-east regions of the USA (such as Alabama, Kentucky, Mississippi, and Tennessee) generally had high mortality rates of respiratory diseases [88]. The West Coast, Texas, and middle east regions experienced high mortality from cardiovascular diseases, whereas the mortality rates were much lower in the northeast and west regions [57,89]. A similar pattern of geographical health disparity was also observed for the mortality rates of neurological disorders and neoplasms [57]. The geographical association between health disparities and air pollutant clusters identified in this study suggests that the regional concentrations of PM_2.5_ mass, PM_10_ speciation, and NONO*_x_*NO*_y_*, analyzed simultaneously, may be an important influencing factor in the real-world setting for regional public health. The high-risk pollution profiles highlight counties where coordinated environmental management and public health actions could yield the largest equity gains. Practitioners could use these profiles to prioritize enhanced monitoring, source-focused emission mitigation (e.g., traffic corridors, industrial point sources, biomass burning), and community exposure-reduction measures such as clean household energy programs, filtration in schools and clinics, and urban greening and heat mitigation.

An ecological study using data reported at the county level is a feasible and labor-intensive approach [57]. This research took advantage of the exhaustive information for county-level demographics and socioeconomic conditions, healthcare provisions, and residual environmental statistics recorded by the US federal offices. It facilitated a thorough control of confounding factors in our statistical analyses. Although it is not available to include most of the USA counties in this study, the samples still provide a relatively broad and representative geographical coverage of the country. This study employed indicators of overall population health, such as life expectancy at birth and mortality rates, rather than more specific health measurements, such as disease incidence and prevalence, which are more likely to be influenced by information bias due to different policies, facilities, and capabilities for disease monitoring, recording, and treatment across the regions. For instance, a higher reported prevalence of bowel cancer in a certain area might actually result from more frequent screenings, improved diagnostic methods, or more effective treatments, subsequently extending survival time. In contrast, life expectancy and mortality statistics, based on death certificates, are less sensitive to such bias. During variable selection, we employed a five-times-repeated ten-fold cross-validation resampling scheme. We identified relatively important variables and conservatively reduced potential covariation through mutual adjustment during screening. These steps help ensure that the resulting clusters reflect stable patterns in pollutant concentrations rather than overfitted solutions. However, the life expectancy in preliminary analyses and subsequent health outcome variables (cause-specific mortality rates) might introduce potential circular reasoning. It was suggested that relatively independent indicators be employed for analysis in future studies. We used backward stepwise regression, entering all exposure variables simultaneously, to identify a parsimonious set for latent class analysis. The exclusion of other key pollutants (e.g., NO_2_, O_3_, and VOCs) suggests that, in our county-average data and conditional on the full set of co-pollutants, PM_2.5_ mass, PM_10_ speciation, and NONO*_x_*NO*_y_* had stronger explanatory power for life expectancy. This should not be interpreted as evidence that the excluded pollutants lack health relevance. Rather, stepwise procedures identify relatively important indicators under mutual adjustment among correlated pollutants. By prioritizing variables with the most stable conditional associations, our approach provides a conservative estimate of the relationship between pollutant-mixture patterns and health outcomes.

In our study, exposure was assessed using monitored data at the group level (i.e., county-level) and therefore is inevitably subject to ecological fallacy. This arises when associations observed at the group level do not accurately reflect relationships at the individual level [90]. County-level exposure assignment may misclassify individual exposures due to spatial and temporal variability and population mobility. Because monitoring networks may not capture within-county spatial variability, county-level values may not provide an unbiased estimate of residents’ exposure. To mitigate this, we employed a database that reflects the exposure characteristics of the primary resident population as comprehensively as possible. While migration rates vary by region, large-scale or targeted inflows are uncommon in relatively stable economies, reducing the likelihood of migration-related exposure bias. Although we adjusted for multiple county-level factors plausibly associated with both pollutant mixtures and health outcomes, unmeasured confounding may remain. For example, socioeconomic deprivation, smoking prevalence, healthcare access, and occupational exposures could influence the associations we observed in this study. These results should be interpreted as identifying geographic pollutant-mixture patterns associated with differences in population health across counties. They are suitable for area-level screening, prioritizing monitoring and emission-control efforts, and generating hypotheses for targeted etiological research. At the individual level, our findings may offer precautionary guidance, particularly for people living in higher-hazard or higher-risk areas. At the same time, we acknowledge that transforming continuous measures into categorical variables prior to latent class analysis results in some loss of information. However, using categorical indicators improves interpretability and yields simpler classes, whereas continuous indicators can complicate clustering—particularly when certain variables exhibit high within-class variability. In the main analysis, Cluster 5 was retained because the five-class solution provided the best fit and separation (lower BIC and higher entropy) and captured a distinct extreme mixture profile. However, its small size leads to wider confidence intervals and greater sensitivity to a small number of counties, so results should be interpreted with caution. In sensitivity analyses excluding Cluster 5, class definitions and associations for the remaining clusters were materially unchanged; merging Cluster 5 with its nearest neighbor yielded similar conclusions. The studied counties varied in their size of residential population, while this study lacked a formal population weighting procedure, which could be a possible source of bias. In this study, we summarized the prolonged exposure to air pollutants using the average level over time; however, other statistics could be used, such as those focusing on the extreme values. Additionally, as previously noted, the findings of our study may be generalized only to the USA, as pollution mixtures, source profiles, regulatory contexts, climate, and urban forms vary across countries. We only included 12 air pollutants, while other potentially important pollutants might be missing. The geographical pattern of air pollutants was determined by LCA with the mentioned selection criteria for the optimal model; however, the use of other statistical approaches may yield slightly different results.

## 5. Conclusions

Our county-level ecological study identified the common geographical patterns of life expectancy-associated air pollutants across the USA. Five distinctive clusters were determined according to the 20-year concentration features of PM_2.5_ mass, PM_10_ speciation, and NONO*_x_*NO*_y_*. In particular, the clusters, characterized by the regions having higher concentrations of these three air pollutants, were associated with a lower life expectancy, higher mortality risks among the middle-aged and elderly populations, and higher mortality rates of several specific causes, including chronic respiratory diseases, cardiovascular diseases, and neoplasms. Our study brings new perspectives on real-world geographical patterns of air pollution to explain health disparities across the USA.

## Figures and Tables

**Table 1:** Descriptive statistics of air pollutant concentrations, health measurements, and socio-demographics at the county level of the USA.

Characteristics, *n* = 699	Median (Interquartile Range)	Mean (Standard Deviation)	Number (%)
Air pollutant concentrations			
PM_2.5_ Mass (μg/m^3^)	9.39 (4.95)	9.03 (3.39)	-
PM_10_ Speciation (μg/m^3^)	15.37 (10.11)	14.80 (7.41)	-
NONO*_x_*NO*_y_* (ppb)	5.95 (7.71)	7.30 (6.84)	-
Health measurements			
Life expectancy, year			
Life expectancy (2014)	78.53 (3.01)	78.44 (2.23)	-
Increase in life expectancy between 1995 and 2014	3.33 (0.01)	3.41 (0.12)	-
Age-specific mortality risk, %			
0–4 years	0.63 (0.22)	0.65 (0.18)	-
5–25 years	0.83 (0.35)	0.87 (0.27)	-
25–44 years	2.77 (1.02)	2.88 (0.84)	-
45–64 years	12.05 (3.68)	12.37 (2.82)	-
65–84 years	50.77 (7.80)	50.52 (5.95)	-
Cause-specific mortality rate, number of deaths/100,000 Population			
Communicable, maternal, neonatal, and nutritional diseases			
HIV/AIDS and tuberculosis	1.12 (1.54)	1.82 (2.05)	-
Diarrhea, lower respiratory, and other common infectious diseases	30.06 (12.10)	30.85 (9.20)	-
Neglected tropical diseases and malaria	0.05 (0.05)	0.06 (0.05)	-
Maternal disorders	0.30 (0.15)	0.33 (0.14)	-
Neonatal disorders	2.94 (1.35)	3.19 (1.17)	-
Nutritional deficiencies	1.35 (0.73)	1.44 (0.66)	-
Other communicable, maternal, neonatal, and nutritional diseases	1.29 (0.44)	1.34 (0.33)	-
Noncommunicable diseases			
Neoplasms	196.77 (37.59)	197.20 (29.60)	-
Cardiovascular diseases	252.63 (67.88)	256.96 (52.17)	-
Chronic respiratory diseases	58.15 (22.06)	59.15 (15.96)	-
Cirrhosis and other chronic liver diseases	17.71 (6.91)	18.87 (7.18)	-
Digestive diseases	15.73 (3.26)	15.63 (2.66)	-
Neurological disorders	99.01 (30.10)	97.94 (21.36)	-
Mental and substance use disorders	13.60 (7.54)	14.84 (6.71)	-
Diabetes, urogenital, blood, and endocrine diseases	56.39 (19.32)	58.24 (15.87)	-
Musculoskeletal disorders	3.16 (1.04)	3.28 (0.86)	-
Other non-communicable diseases	6.22 (1.69)	6.34 (1.37)	-
Injuries			
Transport injuries	16.20 (10.11)	17.89 (8.39)	-
Unintentional injuries	22.29 (6.32)	22.52 (5.41)	-
Self-harm and interpersonal violence	21.44 (8.87)	22.49 (7.24)	-
Forces of nature, war, and legal intervention	0.06 (0.06)	0.08 (0.07)	-
Socio-demographics			
Population characteristics			
Size (per 1000 population), n	108.52 (282.24)	304.38 (624.33)	-
Gender, male %	49.35 (1.38)	49.63 (1.57)	-
Ethnicity, white alone %	87.71 (17.79)	82.78 (14.62)	-
Age, %			
0–9 years	12.35 (2.43)	12.48 (2.06)	-
10–19 years	12.86 (1.86)	12.89 (1.59)	-
20–29 years	12.74 (3.44)	13.30 (3.25)	-
30–39 years	12.13 (2.04)	12.23 (1.75)	-
40–49 years	12.33 (1.86)	12.33 (1.38)	-
50–59 years	14.31 (1.96)	14.25 (1.61)	-
60–69 years	11.36 (3.00)	11.77 (2.53)	-
70–79 years	6.32 (2.31)	6.71 (2.02)	-
≥80years	3.93 (1.53)	4.05 (1.21)	-
Socioeconomics			
Educational level (age ≥ 25), %			
Less than a high school diploma	10.60 (6.00)	11.62 (5.28)	-
A high school diploma only	29.70 (9.15)	29.80 (7.08)	-
Completing some college or an associate’s degree	31.40 (6.95)	31.27 (5.16)	-
A bachelor’s degree or higher	25.40 (13.55)	27.31 (10.64)	-
Median household income (annual, per 1000 US dollars), n	48.57 (15.09)	51.48 (13.53)	-
Unemployment rate, %	6.00 (2.40)	6.28 (2.21)	-
Poverty rate, %	15.50 (7.05)	15.89 (5.51)	-
Healthcare service			
Medical insured population (age < 65), %	86.65 (6.20)	86.63 (4.62)	-
Physicians (per 1000 population), n	1.78 (1.78)	2.15 (1.87)	-
Residential environment and location			
Rural–Urban Continuum Code			
1 (Metro areas, 1 million population or more)	-	-	171 (24.46)
2 (Metro areas, 250 thousand to 1 million population)	-	-	163 (23.32)
3 (Metro areas, population fewer than 250 thousand)	-	-	111 (15.88)
4 (Urban population of 20 thousand or more, adjacent to a metro area)	-	-	50 (7.15)
5 (Urban population of 20 thousand or more, not adjacent to a metro area)	-	-	31 (4.43)
6 (Urban population of 2500 to 19,999, adjacent to a metro area)	-	-	62 (8.87)
7 (Urban population of 2500 to 19,999, not adjacent to a metro area)	-	-	62 (8.87)
8 (Completely rural or less than 2500 urban population, adjacent to a metro area)	-	-	14 (2.00)
9 (Completely rural or less than 2500 urban population, not adjacent to a metro area)	-	-	35 (5.58)
Latitude	38.18 (7.06)	38.82 (5.23)	-
Longitude	−94.92 (24.69)	−94.91 (15.96)	-

**Table 2:** Cluster classification: posterior probability of membership of clusters.

Assigned Cluster, *n* = 699 (%)	Mean Posterior Probability for Each Cluster (Standard Deviation)				
	Cluster 1 (The ‘All Low’ Cluster)	Cluster 2 (The ‘All Medium’ Cluster)	Cluster 3 (The ‘High Particulates’ Cluster)	Cluster 4 (The ‘All High’ Cluster)	Cluster 5 (The ‘Mixed Profile’ Cluster)
Cluster 1 (the ‘all low’ cluster), *n* = 115 (16.5%)	0.66 (0.12)	0.32 (0.08)	0.02 (0.05)	0.00 (0.00)	0.00 (0.00)
Cluster 2 (the ‘all medium’ cluster), *n* = 285 (40.8%)	0.02 (0.03)	0.88 (0.14)	0.06 (0.10)	0.03 (0.08)	0.02 (0.05)
Cluster 3 (the ‘high particulates’ cluster), *n* = 152 (21.7%)	0.01 (0.01)	0.13 (0.13)	0.84 (0.13)	0.01 (0.01)	0.01 (0.01)
Cluster 4 (the ‘all high’ cluster), *n* = 136 (19.4%)	0.00 (0.00)	0.06 (0.06)	0.01 (0.00)	0.93 (0.06)	0.00 (0.00)
Cluster 5 (the ‘mixed profile’ cluster), *n* = 11 (1.6%)	0.00 (0.00)	0.01 (0.01)	0.08 (0.02)	0.00 (0.00)	0.91 (0.01)

**Table 3:** Associations of air pollutant clusters with life expectancy and change in life expectancy with confounding adjustment.

Variables in Multivariate Regression Analysis	Life Expectancy, 2014 Adjusted R^2^ = 0.67		Change in Life Expectancy, 1995–2014 Adjusted R^2^ = 0.45	
	Coefficient	99.5% Confidence Intervals	Coefficient	99.5% Confidence Intervals
Clusters of air pollutants				
Cluster 1 (the ‘all low’ cluster)	0 (referent)	-	0 (referent)	-
Cluster 2 (the ‘all medium’ cluster)	**−0.36**	**(−0.80, −0.08)**	−0.25	(−0.56, 0.05)
Cluster 3 (the ‘high particulates’ cluster)	**−0.79**	**(−1.32, −0.27)**	**−0.40**	**(−0.77, −0.04)**
Cluster 4 (the ‘all high’ cluster)	**−0.95**	**(** **−1.50, −0.39)**	**−0.46**	**(** **−0.84, −0.07)**
Cluster 5 (the ‘mixed profile’ cluster)	−0.83	(−2.02, 0.36)	0.33	(−0.50, 1.15)
Population size (per 1000 population), n	**0.0006**	**(0.0003, 0.0008)**	**0.0004**	**(0.0002, 0.0006)**
Gender, male %	0.11	(−0.01, 0.22)	**0.12**	**(0.05** **, 0.20)**
Ethnicity, white alone %	**0.04**	**(0.02, 0.05)**	**−0.02**	**(−0.03** **, −0.01)**
Education level, bachelor’s degree %	**−0.12**	**(−0.15, −0.09)**	**−0.05**	**(−0.07** **, −0.03)**
Median household income (per 1000 US dollars), n	**0.04**	**(0.02, 0.06)**	0.01	(−0.002, 0.029)
Unemployment rate, %	**0.08**	**(0.004, 0.16)**	**0.16**	**(0.10** **, 0.21)**
Poverty rate, %	**−0.10**	**(−0.15, −0.04)**	−0.03	(−0.07, 0.01)
Insured population, %	0.02	(−0.02, 0.06)	**−0.03**	**(−0.06** **, −0.0008)**
Physicians (per 1000 population), n	0.06	(−0.04, 0.16)	**0.11**	**(0.04** **, 0.18)**
Rural–Urban Continuum Code				
1	0 (referent)	-	0 (referent)	-
2	0.39	(−0.06, 0.84)	**−0.39**	**(−0.70** **, −0.08)**
3	0.49	(−0.04, 1.03)	**−0.49**	**(−0.86** **, −0.11)**
4	0.62	(−0.05, 1.28)	−0.17	(−0.63, 0.30)
5	0.21	(−0.58, 1.01)	−0.51	(−1.07, 0.04)
6	**0.80**	**(0.13, 1.46)**	−0.24	(−0.71, 0.22)
7	0.36	(−0.31, 1.04)	−0.37	(−0.84, 0.10)
8	0.90	(−0.20, 2.01)	0.01	(−0.76, 0.78)
9	**0.98**	**(0.15, 1.82)**	−0.14	(−0.72, 0.44)
Latitude	0.01	(−0.02, 0.05)	**0.03**	**(0.002** **, 0.05)**
Longitude	0.003	(−0.01, 0.01)	**0.01**	**(0.01** **, 0.02)**

## Data Availability

1. All air pollutant data were publicly accessible from the US Environmental Protection Agency, Air Quality System Data Mart [52]. 2. County-level population health metrics, including life expectancy at birth and age-specific mortality risks, were extracted from the database provided by the Institute for Health Metrics and Evaluation [56]. 3. County-level 21 mutually exclusive age-standardized cause-specific mortality rates were extracted from the database provided by the Institute for Health Metrics and Evaluation [58]. 4. County-level population size, gender, age, and ethnic distribution data were extracted from the databases provided by the USA Bureau of the Census [63]. 5. County-level educational level data were extracted from the databases provided by the USA Bureau of the Census [64]. 6. County-level annual median household income and poverty rate data were extracted from the databases provided by the USA Bureau of the Census [65]. 7. County-level unemployment rate data were extracted from the databases provided by the USA Bureau of Labor Statistics [59]. 8. County-level insured population rates were extracted from the databases provided by the USA Bureau of the Census [61]. 9. County-level representative latitude and longitude coordinates were extracted from the databases provided by the USA Bureau of the Census [62]. 10. County-level numbers of physicians were extracted from the database provided by the USA Health Resources and Services Administration [60]. 11. The Rural–Urban Continuum Code was extracted from the database provided by the USA Department of Agriculture [66].

## Supplementary Materials

The following supporting information can be downloaded at: https://www.mdpi.com/article/10.3390/life16010177/s1, Table S1: Measured parameters, units, and classification of all studied air pollutants; Table S2: Sources of population characteristics data, socio-demographic data, and health outcome data; Table S3: Results of twenty repeats of ten-fold cross-validation resampling scheme for assessing the life expectancy-associated air pollutants; Table S4: Statistical assessment of the optimal number of clusters from latent class analysis models based on ordinal data after multiple imputations; Table S5: County-level statistics of air pollutant concentrations, and health measurements stratified by latent class analysis-derived clusters; Figure S1: Flowchart of planned statistical approach and analysis; Figure S2: Geographical distribution of counties included in the final dataset across the USA (*n* = 699); Figure S3: Geographical distribution of air monitors used in the statistical analysis across the USA (*n* = 2453).
