# Supplementary material for: Spatial Cluster of Air Pollutants and Its Association with Life Expectancy, Age-Specific Mortality Risk, and Cause-Specific Mortality Rate: A County-Level Ecological Study Across the USA
Source: Life (Basel). 2026 Jan 21;16(1):177. doi: 10.3390/life16010177 (PMC12842859; doi:10.3390/life16010177)
Supplement: Supplementary file 1 [file life-16-00177-s001.zip › life-4076120-supplementary.pdf]

Table of contents

Table S1 Measured parameters, units, and classification of all studied air pollutants

Table S2 Sources of population characteristics data, socio-demographic data, and health outcome data

Table S3 Results of twenty repeats of ten-fold cross-validation resampling scheme for assessing the life expectancy-associated air pollutants

Table S4 Statistical assessment of the optimal number of clusters from latent class analysis models based on ordinal data after multiple imputations

Table S5 County-level statistics of air pollutant concentrations, and health measurements stratified by latent class analysis-derived clusters

Figure S1 Flowchart of planned statistical approach and analysis

Figure S2 Geographical distribution of counties included in the final dataset across the USA (n=699)

Figure S3 Geographical distribution of air monitors used in the statistical analysis across the USA (n=2453)

Table S1. Measured parameters, units, and classification of all studied air pollutants

Air pollutant	Measured parameter	Units
Criteria gases		
Ozone	Ozone	Parts per million
CO	CO	Parts per million
SO_2	SO_2	Parts per billion
NO_2	NO_2	Parts per billion
Particulates		
$PM_{2.5}$ / PM_{10} mass	Total mass concentration of $PM_{2.5}$ / PM_{10}	Micrograms/cubic meter
$PM_{2.5}$ / PM_{10} speciation	Multiple components of the $PM_{2.5}$/ PM_{10}, which are 1) several elements including crustal or soil-related (i.e., Si, Al, Ti, Ca, Fe) and other common elements (i.e., K, Cl, P, Mg, Cr), 2) soluble ions including Nitrate, sulfate, sodium, potassium, chloride, ammonium, as well as 3) biological material and 4) fly ash, and 5) carbon including organic and elemental carbon and carbonate carbon	Micrograms/cubic meter
Toxics, precursors, and lead		
Hazardous air pollutants (HAPs)	Arsenic	Micrograms/cubic meter
Volatile organic compounds (VOCs)	Total non-methane organic compound (NMOC)	Parts per billion Carbon
$NONO_xNO_y$	Nitric oxide	Parts per billion
Lead	Lead	Micrograms/cubic meter

Table S2. Sources of population characteristics data, socio-demographic data, and health outcome data

Data	Institution of the Data Sources	Details
Population size (per 1,000 population), gender, age, and ethnic distribution, county-level	USA Bureau of the Census	County characteristics resident population estimates, 2014 data
Educational level (aged ≥25), county-level	USA Bureau of the Census	American community survey 5-year average county-level estimates, 2014 data
Annual median household income, county-level	USA Bureau of the Census	Small Area Income and Poverty Estimates Program, 2014 data
Unemployment rate, county-level	USA Bureau of Labor Statistics	Local area unemployment statistics, 2014 data
Poverty rate, county-level	USA Bureau of the Census	Small Area Income and Poverty Estimates Program, 2014 data
Insured population (aged <65) rate, county-level	USA Bureau of the Census	Small Area Health Insurance Estimates Program, 2014 data
Physicians' number (per 1,000 population), county-level	USA Health Resources and Services Administration	Area Health Resources File: Total active non-federal MDs, 2014 data
Rural-urban continuum code	USA Department of Agriculture	Economic Research Service, 2014 data
Latitude and longitudes, county-level	USA Bureau of the Census	Representative county-level latitude and longitude coordinates
Life expectancy at birth, county-level	Institute for Health Metrics and Evaluation	Life expectancy at birth, 1995-2014 data
Age-specific mortality risk, county-level	Institute for Health Metrics and Evaluation	The age-specific mortality risks (2014 data) are provided as the age groups of 0-4, 5-24, 25-44, 45-64, and 65-84
Cause-specific mortality rate, county-level	Institute for Health Metrics and Evaluation	The cause-specific mortality rates (2014 data) include 21 mutually exclusive causes of death

Table S3. Results of twenty repeats of ten-fold cross-validation resampling scheme for assessing the life expectancy-associated air pollutants

Repeated time	NO_2	$PM_{2.5}$ mass	PM_{10} mass	PM_{10} speciation	$NONO_xNO_y$
1	√	√	×	√	√
2	×	√	×	√	√
3	√	√	×	√	√
4	×	√	×	√	√
5	×	√	×	√	√
6	√	√	×	√	√
7	×	√	×	√	√
8	×	√	×	√	√
9	×	√	×	√	√
10	√	√	×	√	√
11	√	√	×	√	√
12	√	√	×	√	√
13	×	√	×	√	√
14	×	√	×	√	√
15	×	√	×	√	√
16	×	√	×	√	√
17	×	√	×	√	√
18	×	√	×	√	√
19	√	√	×	√	√
20	√	√	√	√	√
**Total**	**8**	**20**	**1**	**20**	**20**

√ and × represent air pollutants that were and were not selected and enrolled in the optimal model of the backward selection results, respectively.

Table S4. Statistical assessment of the optimal number of clusters from latent class analysis models based on ordinal data after multiple imputations

Model	LL statistics	BIC_{LL}	$CAIC_{LL}$	Bootstrapping p-value
1 cluster (H_0)	-2072.36	4184.03	4190.03	<0.0001
2 cluster	-2051.28	4168.05	4178.05	<0.0001
3 cluster	-2029.11	4149.91	4163.91	<0.0001
4 cluster	-2017.60	4153.10	4171.10	0.0003
5 cluster †	-2011.81	4167.72	4189.72	0.0013
6 cluster	-2012.39	4195.08	4221.08	Non-significant
7 cluster	-2005.03	4206.55	4236.55	Non-significant
8 cluster	-2003.71	4230.11	4264.11	Non-significant
9 cluster	-2003.66	4256.20	4294.20	Non-significant
10 cluster	-2003.62	4282.32	4324.32	Non-significant

LL, Log-likelihood Statistics; BIC_{LL}, Bayes Information Criterion based on Log-likelihood Statistics; $CAIC_{LL}$, Consistent Akaike's Information Criterion based on Log-likelihood Statistics. †The optimal model.

Table S5. County-level statistics of air pollutant concentrations, and health measurements stratified by latent class analysis-derived clusters

Characteristics, *n*=699	Cluster 1 (the 'all low' cluster)	Cluster 2 (the 'all medium' cluster)	Cluster 3 (the 'high particulates' cluster)	Cluster 4 (the 'all high' cluster)	Cluster 5 (the 'mixed profile' cluster)
Air pollutant concentrations					
$PM_{2.5}$ Mass (μg/m^3)	4.67 (1.73)	7.86 (2.40)	10.98 (1.87)	12.83 (1.51)	10.92 (2.30)
PM_{10} Speciation (μg/m^3)	8.94 (6.03)	12.98 (6.00)	18.99 (6.30)	19.83 (6.22)	2.84 (4.21)
$NONO_xNO_y$ (ppb)	2.67 (3.14)	5.74 (5.41)	8.65 (8.44)	12.81 (6.02)	9.03 (0.46)
Health measurements					
Life expectancy, year					
Life expectancy (2014)	79.33 (1.84)	78.91 (2.14)	77.81 (2.26)	77.40 (2.03)	78.49 (3.09)
Change in life expectancy between 1995 and 2014	3.50 (0.81)	3.41 (1.01)	3.34 (1.40)	3.35 (1.44)	4.40 (2.48)
Age-specific mortality risk, %					
0 – 4 years	0.62 (0.16)	0.61 (0.16)	0.70 (0.20)	0.72 (0.17)	0.64 (0.25)
5 – 25 years	0.90 (0.30)	0.84 (0.27)	0.87 (0.26)	0.89 (0.25)	0.81 (0.33)
25 – 44 years	2.79 (0.82)	2.72 (0.81)	3.03 (0.90)	3.14 (0.78)	2.96 (0.89)
45 – 64 years	11.14 (2.03)	11.71 (2.62)	13.23 (2.94)	13.80 (2.71)	12.80 (3.56)
65 – 84 years	47.3 (5.09)	49.47 (5.72)	52.35 (5.64)	53.47 (5.19)	49.88 (9.05)
Cause-specific mortality rate, number of deaths per 100,000 Population					
Communicable, maternal, neonatal, and nutritional diseases					
HIV/AIDS and tuberculosis	2.65 (2.48)	1.38 (1.33)	2.96 (2.86)	2.61 (2.68)	2.96 (2.86)
Diarrhea, lower respiratory and other common infectious diseases	36.2 (8.58)	28.95 (8.80)	31.9 (8.75)	33.59 (8.86)	31.9 (8.75)
Neglected tropical diseases and malaria	0.05 (0.03)	0.07 (0.04)	0.05 (0.03)	0.06 (0.03)	0.05 (0.03)
Maternal disorders	0.39 (0.15)	0.30 (0.12)	0.32 (0.12)	0.38 (0.18)	0.32 (0.12)
Neonatal disorders	3.9 (1.16)	2.87 (0.84)	3.2 (1.49)	3.63 (1.46)	3.20 (1.49)
Nutritional deficiencies	1.51 (0.70)	1.36 (0.66)	1.08 (0.72)	1.57 (0.67)	1.08 (0.72)
Other communicable, maternal, neonatal, and nutritional diseases	1.39 (0.31)	1.28 (0.30)	1.44 (0.35)	1.46 (0.35)	1.44 (0.35)
Noncommunicable diseases					
Neoplasms	211.69 (25.61)	192.21(27.59)	198.25 (39.16)	206.12 (30.53)	198.25 (39.16)

Cardiovascular diseases	285.77 (49.78)	247.17 (47.59)	279.43 (72.45)	272.77 (52.29)	279.43 (72.45)
Chronic respiratory diseases	62.17 (16.84)	58.11 (15.51)	56.48 (21.97)	61.68 (16.48)	56.48 (21.97)
Cirrhosis and other chronic liver diseases	18.63 (4.34)	18.56 (7.46)	18.48 (7.03)	18.65 (5.08)	18.48 (7.03)
Digestive diseases	15.78 (2.62)	15.30 (2.66)	14.91 (3.86)	15.93 (2.52)	14.91 (3.86)
Neurological disorders	103.34 (20.52)	96.32 (20.11)	84.32 (22.97)	103.98 (20.84)	84.32 (22.97)
Mental and substance use disorders	16.35 (6.84)	14.17 (6.01)	16.34 (6.57)	14.2 (5.99)	16.34 (6.57)
Diabetes, urogenital, blood and endocrine diseases	65.19 (14.74)	55.19 (14.57)	52.14 (15.96)	63.04 (17.12)	52.14 (15.96)
Musculoskeletal disorders	3.05 (0.70)	3.27 (0.94)	2.60 (0.82)	3.22 (0.66)	2.60 (0.82)
Other non-communicable diseases	6.66 (1.48)	6.08 (1.27)	6.03 (2.14)	6.69 (1.41)	6.03 (2.14)
Injuries					
Transport injuries	16.74 (7.06)	17.62 (8.72)	15.32 (10.62)	16.8 (6.88)	15.32 (10.62)
Unintentional injuries	21.71 (4.77)	22.07 (5.16)	17.76 (6.82)	22.13 (4.70)	17.76 (6.82)
Self-harm and interpersonal violence	22.16 (5.47)	21.63 (7.22)	20.49 (7.03)	22.23 (6.98)	20.49 (7.03)
Forces of nature, war, and legal intervention	0.06 (0.05)	0.07 (0.08)	0.07 (0.05)	0.07 (0.07)	0.07 (0.05)

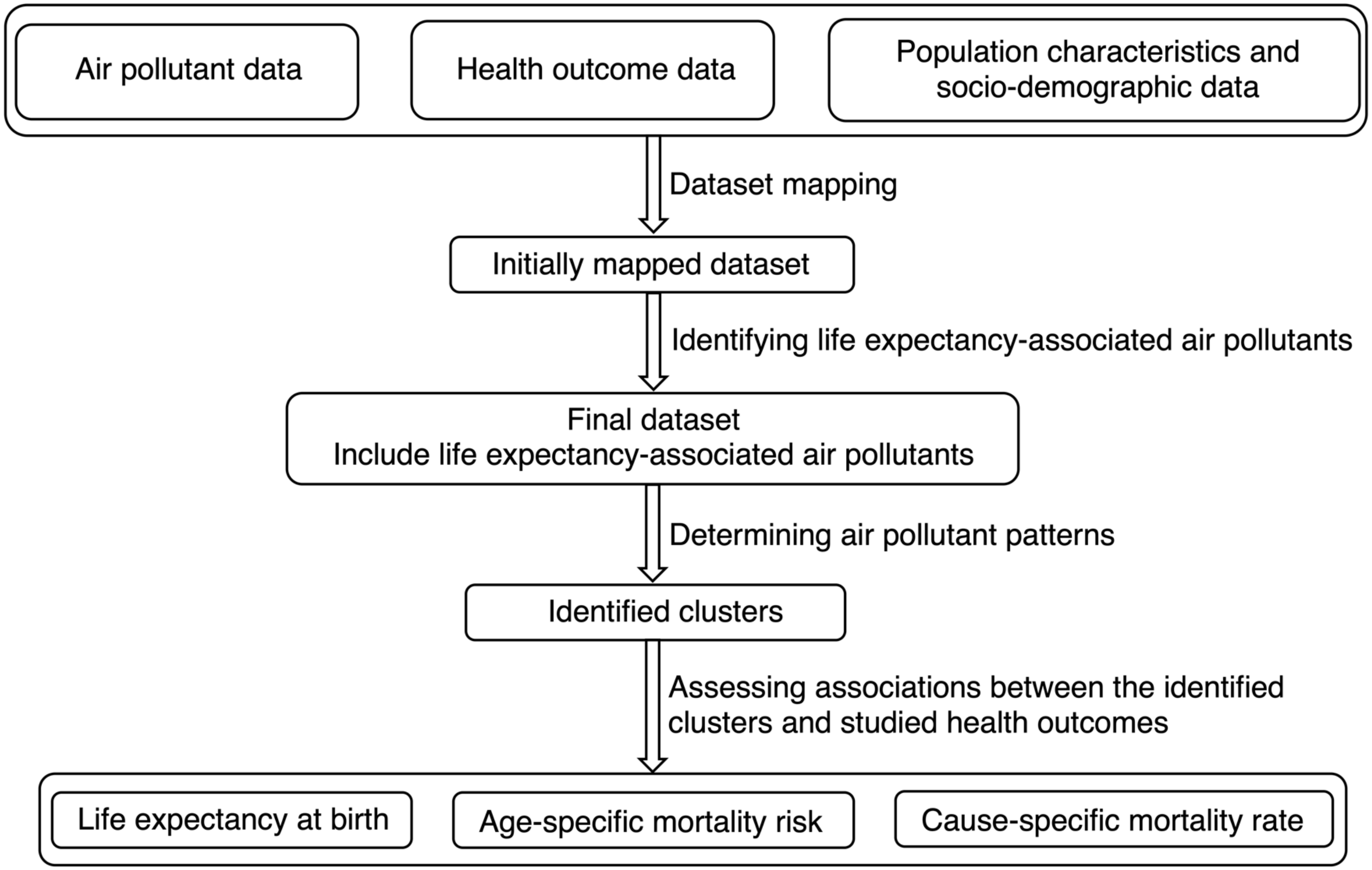

Figure S1. Flowchart of planned statistical approach and analysis

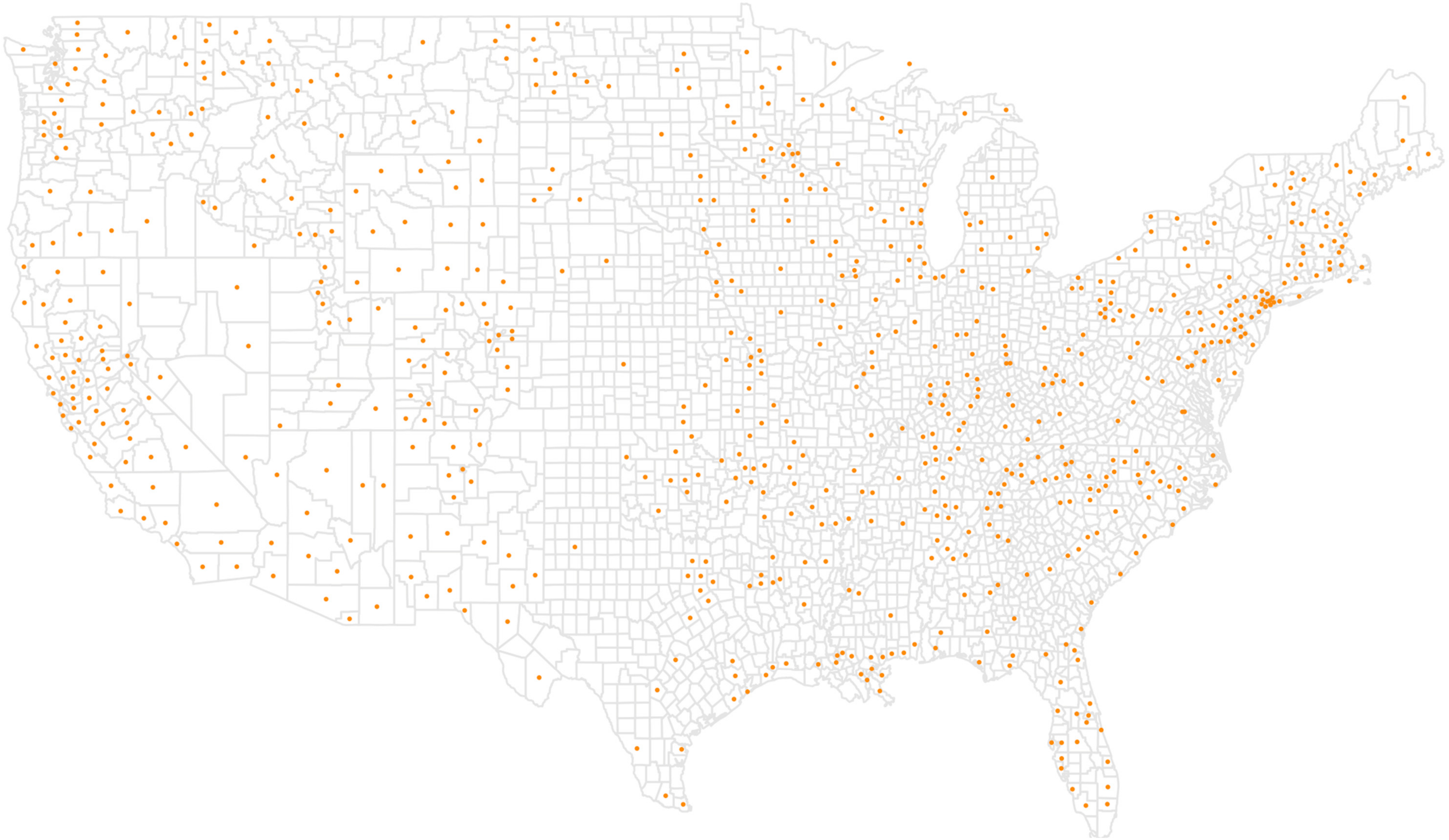

Figure S2. Geographical distribution of counties included in the final dataset across the USA (n=699)

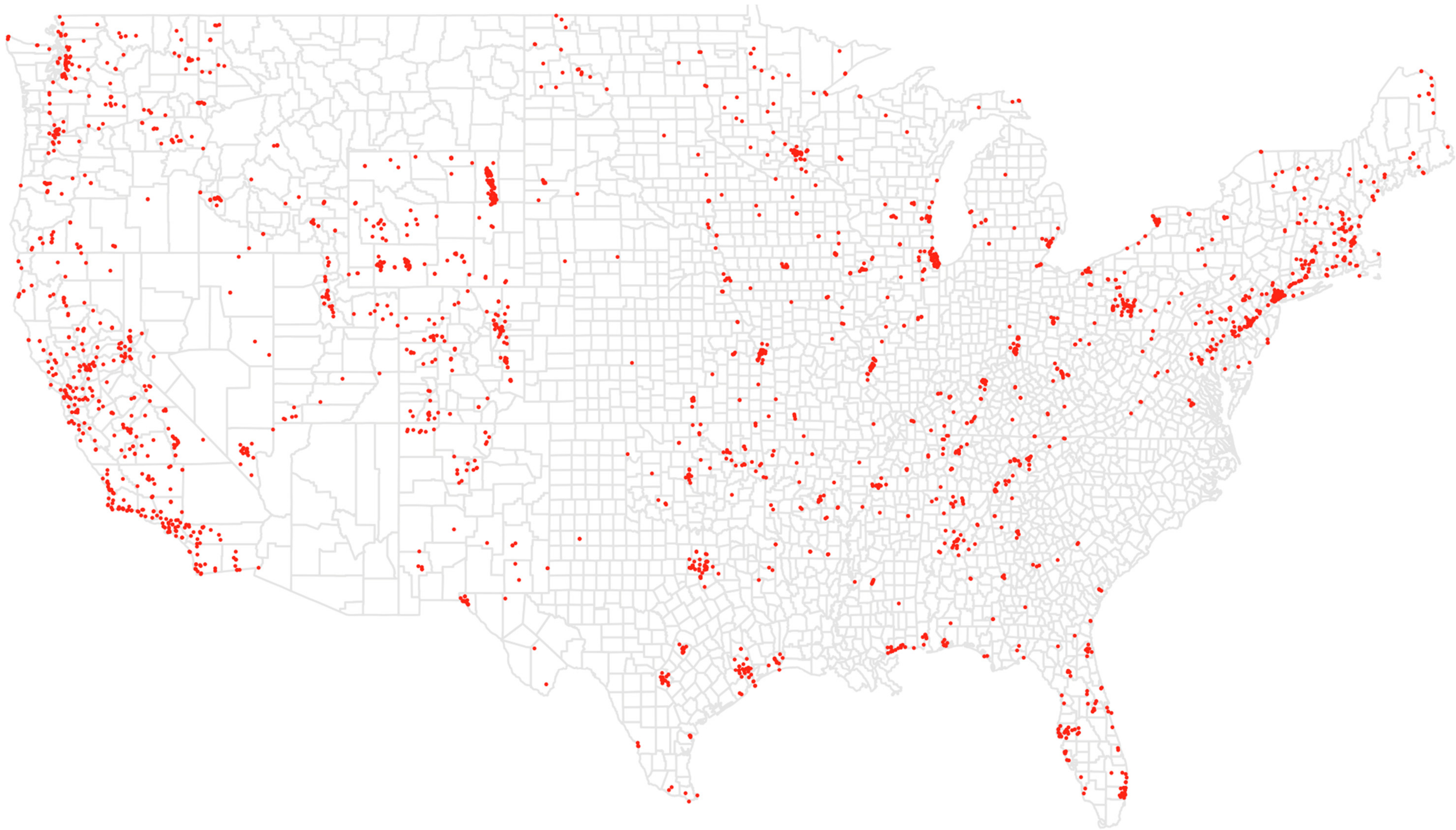

Figure S3. Geographical distribution of air monitors used in the statistical analysis across the USA (n=2453)
